# Strigolactone insensitivity affects differential shoot and root transcriptome in barley

**DOI:** 10.1007/s13353-024-00885-w

**Published:** 2024-06-14

**Authors:** Magdalena Korek, R. Glen Uhrig, Marek Marzec

**Affiliations:** 1 Faculty of Natural Sciences, Institute of Biology, Biotechnology and Environmental Protection, University of Silesia in Katowice, Jagiellonska 28, 40-032 Katowice, Poland; 2 Department of Biological Sciences, University of Alberta, 11455 Saskatchewan Drive, Edmonton, AB T6G 2E9 Canada

**Keywords:** Barley, DWARF14, *Hordeum vulgare*, Root, Shoot, Strigolactones, Transcriptome

## Abstract

Strigolactones (SLs) are plant hormones that play a crucial role in regulating various aspects of plant architecture, such as shoot and root branching. However, the knowledge of SL-responsive genes and transcription factors (TFs) that control the shaping of plant architecture remains elusive. Here, transcriptomic analysis was conducted using the SL-insensitive barley mutant *hvd14.d* (carried mutation in SL receptor DWARF14, HvD14) and its wild-type (WT) to unravel the differences in gene expression separately in root and shoot tissues. This approach enabled us to select more than six thousand SL-dependent genes that were exclusive to each studied organ or not tissue-specific. The data obtained, along with *in silico* analyses, found several TFs that exhibited changed expression between the analyzed genotypes and that recognized binding sites in promoters of other identified differentially expressed genes (DEGs). In total, 28 TFs that recognize motifs over-represented in DEG promoters were identified. Moreover, nearly half of the identified TFs were connected in a single network of known and predicted interactions, highlighting the complexity and multidimensionality of SL-related signalling in barley. Finally, the SL control on the expression of one of the identified TFs in HvD14- and dose-dependent manners was proved. Obtained results bring us closer to understanding the signalling pathways regulating SL-dependent plant development.

**Supplementary Information:**

The online version contains supplementary material available at 10.1007/s13353-024-00885-w.

## Introduction

Strigolactones (SLs) are phytohormones involved in the control of plant architecture, including shoot branching, plant height (Gomez-Roldan et al. 2008; Umehara et al. 2008) as well as root elongation and branching (Koltai 2011). Grafting studies revealed that SLs may be synthesized in roots (Beveridge 2000; Booker et al. 2005) and transported to the aboveground organs via SL-specific transporters (Kretzschmar et al. 2012). On the other hand, SLs are also secreted via roots to the rhizosphere, where they act as signal molecules in communication with other organisms, such as bacteria, fungi and other plants (Kee et al. 2023). Moreover, studies from recent years indicate that SLs play critical functions in the plant response to stresses, especially abiotic ones (Yoneyama et al. 2012). Plants adapt to changing environmental conditions via SL-mediated modulation of underground and aboveground organ development (Trasoletti et al. 2022). Under control conditions, SLs inhibit the shoot branching (Gomez-Roldan et al. 2008; Umehara et al. 2008). Thus, mutants deficient in SL biosynthesis or signalling exhibit a bushy phenotype. In contrast, the application of SLs reduces shoot branching (reviewed by Kelly et al. 2023). The SL receptor D14 (DWARF14) recognizes the SL molecule, which changes the receptor conformation to facilitate SL signalling complex assembly (Marzec and Brewer 2019). This complex binds the SL repressor D53 (DWARF53), which undergoes proteasomal degradation in an SL-dependent manner (Zhou et al. 2013) to activate SL-dependent transcription factors (TFs) and their target genes. The key SL-dependent TF involved in the regulation of shoot branching is BRC1 (BRANCHED1). Initially, BRC1 was identified in *Arabidopsis* (*Arabidopsis thaliana)* (Aguilar-Martínez et al. 2007), and its ortholog TB1 (TEOSINTE BRANCHED1) was characterized in monocotyledons rice (*Oryza sativa*) (Takeda et al. 2003) and maize (*Zea mays*) (Doebley et al. 1997). It was shown that *BRC1/TB1* expression is limited to the axillary buds, which negatively correlates with bud outgrowth (Takeda et al. 2003; Aguilar-Martínez et al. 2007). The bushy phenotype of *brc1* mutants in *Arabidopsis* and pea (*Pisum sativum*) cannot be reversed by SL treatment, indicating the BRC1 action downstream of SLs (Brewer et al. 2009). Moreover, exogenous SLs elevate the expression of *BRC1* in wild-type (WT) plants, while *BRC1* expression is reduced in SL mutants (Dun et al. 2012), clearly showing that SLs regulate shoot branching via BRC1/TB1. On the other hand, the outgrowth of axillary buds depends on the export of auxin from buds via PIN-FORMED (PIN) protein efflux carrier proteins (Wiśniewska et al. 2006). It was shown that SL biosynthesis mutants exhibited increased PIN1 abundance and therefore increased auxin transport, which results in the highly branched phenotype (Bennett et al. 2006). At the same time, SL treatment disturbs the cellular localization of PIN1 (Shinohara et al. 2013). Thus, SLs control shoot architecture via modulation of BRC1/TB1 activity and control of auxin transport. To date, the role of SLs in inhibiting axillary bud outgrowth was the best characterized function of SLs in plants. Moreover, the results obtained for different species are consistent and reproducible (Kelly et al. 2023).

The role of SLs in shaping root architecture was proposed in 2011 based on studies in *Arabidopsis* (Kapulnik et al. 2011; Ruyter-Spira et al. 2011). Both SL biosynthesis and signalling mutants developed a higher number of lateral roots compared to the WT, and SL treatment reduced the lateral root number in WT and SL biosynthesis mutants, but not in SL signalling mutant (Kapulnik et al. 2011; Ruyter-Spira et al. 2011). The inhibitory SL effect on lateral root density was also observed in other species, such as *Lotus japonicus* (Liu et al. 2013), *Medicago truncatula* (De Cuyper et al. 2015) and barley (*Hordeum vulgare*) (Marzec et al. 2016). SL biosynthesis and signalling mutants in rice developed a similar number of lateral roots to the WT (Arite et al. 2012); the SL treatment still reduced lateral root density in WT rice plants (Sun et al. 2014). Reduction of lateral root density in rice after SL application was linked with decreased expression of several genes encoding PINs and inhibition of auxin transport from shoot to root (Arite et al. 2012; Sun et al. 2014). Moreover, an elevated auxin concentration was observed in the root tissue of rice SL biosynthesis mutant (*Osd10/17*) (Sun et al. 2014, 2015). When the standard concentration of auxin is present in the *Arabidopsis* root, SLs regulate the cellular localization of PINs and thus repress the expression of auxin-dependent genes and reduce the lateral root number (Ruyter-Spira et al. 2011; Zhang et al. 2020a, b). Conversely, increased auxin concentration in root SLs promotes the development of lateral roots (Ruyter-Spira et al. 2011; Mayzlish-Gati et al. 2012). In response to various stresses, such as nutrient deficiency, drought, salinity, or increased heavy metal concentration, the impact of SLs on root system development became more enigmatic (Marzec and Melzer 2018; Sun et al. 2022). Hence, the role of SLs in root development is much more complicated than in the case of shoots, and it is also affected by many factors, such as growing conditions or plant age.

In recent years, significant progress in understanding the function of SLs has been made thanks to high-throughput comparative analyses of SL mutants or SL-treated vs. untreated plants. Wang and co-workers identified 401 SL-dependent genes in *Arabidopsis*, including three TFs involved in SL signal transduction. Besides well-known BRC1, the TFs which control anthocyanin biosynthesis (PRODUCTION OF ANTHOCYANIN PIGMENT 1, PAP1) or leaf development (TCP DOMAIN PROTEIN 1, TCP1) were found to be under the control of SLs (Wang et al. 2020). Analyses of transcriptome changes mediated by SLs or auxin (indole-3-acetic acid; IAA) in tomato (*Solanum lycopersicum*) shoots revealed a higher number of differentially expressed genes (DEG) after auxin application. However, among the smaller number of genes whose expression was altered by SL treatment, the upregulated genes of the auxin signalling pathway were found, indicating the crosstalk between SLs and auxin in tomato (Zhan et al. 2018). At the same time, melon (*Cucumis melo*) root transcriptome analyses revealed the crosstalk between SLs and auxin in promoting adventitious root growth (Li et al. 2023). Root transcriptome was also investigated for rice WT and SL biosynthesis mutant in response to phosphorus starvation and SL application. Those experiments uncovered the enzyme METHYL TRANSFERASE (Os01g0700300) to be involved in SL biosynthesis (Haider et al. 2023), while treatment of apple rootstock M26 with SL synthetic analogue GR24 or SL inhibitor Tis108 revealed SLs to promote adventitious shoot formation, facilitating the identification of more than 10,000 potentially SL-responsive genes (Asghar et al. 2022). Finally, the role of SLs in plant response to drought was investigated via transcriptome analyses in various species, including *Arabidopsis* (Li et al. 2020a; Korwin Krukowski et al. 2023), rice (Yoo et al. 2017) and barley (Daszkowska-Golec et al. 2023). Based on these results, the molecular basis of the role of SL in response to drought stress was described, including interaction with abscisic acid, increased synthesis and deposition of waxes or ROS scavenging. Moreover, the first SL-dependent TFs that can mediate the adaptation of plants to water deficit have been identified.

In the presented study, we use a previously characterized barley line *hvd14.d*, which is SL-insensitive due to the mutation in SL receptor HvD14 (Marzec et al. 2016), to investigate the role of SL in the control of shoots and roots architecture. The *hvd14.d* line has been characterized to exhibit the SL-insensitivity phenotype: semi-dwarf and highly branched shoot, as well as a root system composed of shorter seminal roots, which developed a more significant number of lateral roots, compared to the WT (Marzec et al. 2016). Moreover, *hvd14.d* mutant is more sensitive to drought (Marzec et al. 2020), which was also observed for SL-insensitive mutants in other species (Haider et al. 2018; Li et al. 2020a; Korwin Krukowski et al. 2023). Here, we use *hvd14.d* line to uncover tissue-specific SL-dependent mechanisms disturbed in this line, which affects barley shoot and root phenotype. The transcriptomic differences between *hvd14.d* and its WT were investigated separately for the shoot and root tissue. That approach allowed us to dissect the SL-related regulatory mechanisms specific to each investigated organ and those not tissue-specific.

## Materials and method

*Plant material*, *growth conditions and hormone treatment*

Two genotypes were used in the described studies: wild-type variety Sebastian and *hvd14.d* mutant obtained after chemical mutagenesis (Szurman-Zubrzycka et al. 2018). Mutant *hvd14.d* is insensitive to strigolactones due to the mutation in strigolactone receptor HvD14 (Marzec et al. 2016)*.*

For the RNAseq experiment, plants were grown in hydroponic conditions for up to 21 days. Six plants were placed in the 1.5 l container filled with ½ Hoagland solution (Hothem et al. 2003). The medium was replaced every week. Plants were placed in the greenhouse under a 20/18 °C day/night, 16/8 photoperiod and 420 μE m^−2^ s^−1^ light intensity. Total root length, lateral root length and density were determined using an Epson scanner and WINRHIZO software (Regent Instruments Inc.).

For the spraying experiments, five plants were sown in the pot (7.5 × 7.5 × 10 cm) filled with soil garden. Two-week-old seedlings were sprayed with 1 or 10 µM of GR24^5DS^ (StrigoLab, Turin, Italy). Control plants were sprayed with a mock solution (0.01% acetone). Tissue for RT-qPCR analyses were collected from plants before treatment and after 0.5, 1 and 3 h after treatment.

### RNA isolation and RNA sequencing

For RNA-isolation analyses, plant tissue (shoot and root) was collected in four biological replicates, each containing tissue from four seedlings. Samples were frozen immediately in liquid nitrogen; RNA was isolated using the mirVana miRNA Isolation Kit (ThermoFisher Scientific, catalogue number: AM1560). Library construction and sequencing (150-nt paired-end reads) on Illumina NovaSeq™ 6000 platforms were performed by the Novogene Genomics Service (Cambridge, United Kingdom). The Novogene Genomics Service also provided basic data analysis by applying their RNAseq pipeline. Genes with adjusted *p*-value < 0.05 and log_2_FC ≥ 1 or ≤ −1 were considered differentially expressed.

### RT-qPCR

RNA was extracted as described previously, in four biological replicates, each containing tissue from five seedlings. RevertAid First Strand cDNA Synthesis Kit (Product No. K1621, Life Technologies) was used for cDNA synthesis. Diluted cDNA (1:4, cDNA:water) was used for RT-qPCR reactions performed using LightCycler FastStart DNA Master SYBR Green (Product No. 12239264001, Roche) and LightCycler 480 Instrument II (Roche). Relative expression level of HORVU.MOREX.r2.1HG0041130 (F: AGGGACCTGGAGTGGTTCTT, R: AACACCAGCGTCTTCCTGAC) calculated and normalized to the internal control, the EF1 gene (Elongation factor 1-α; F: CCCTCCTCTTGGTCGTTTTG; R: ATGACACCAACAGCCACAGTTT). Data were analyzed using LinRegPCR (Ramakers et al. 2003). Four biological replicates were analyzed for each time point in two technical replicates. A relative expression level was presented to control, fixed as 1. Data are presented as mean ± SE of 2^−∆∆Ct^ in each case. Statistical analyses were performed using the *t*-test (*p < .05; **p < .01; ***p < .001).

### Gene ontology

Gene ontology (GO) enrichment was performed using ShinyGO 0.77 tool (http://bioinformatics.sdstate.edu/go/) (Ge et al. 2020). Gene Lists from Supplementary Table 1 were used as a query, and the following settings were used: FDR cutoff: 0.05, pathways to show: 20, min. pathway size: 2, max. pathway size: 2000. Treemaps for the GO biological process were prepared using the ReviGO tool (http://revigo.irb.hr/) (Supek et al. 2011). Plot size was adjusted to the Log10 *p*-value of the GO-term enrichment. Only the biological process GO category was used. The *p*-value of each GO term was obtained using the AgriGO tool (http://systemsbiology.cau.edu.cn/agriGOv2) (Tian et al. 2017).

### TF prediction and promoter analysis

Amino acid sequences of all identified DEG were obtained using BioMart Ensemble Plant (http://plants.ensembl.org/info/data/biomart/index.html) v56 from ‘Hordeum vulgare TRITEX genes (Morex_V2_scaf)’ datasets. Those sequences were used as a query in the ‘Transcription Factor Prediction’ tool from PlanRegMap (http://planttfdb.gao-lab.org/prediction.php) (Tian et al. 2019). As a result, probable TFs (with MLOC IDs) and their *Arabidopsis* orthologs were obtained (Supplementary Data 4).

Promoter sequence (1500 bp before START codon) of all identified DEG were obtained using BioMart Ensamble Plant (http://plants.ensembl.org/info/data/biomart/index.html) v56 from ‘Hordeum vulgare TRITEX genes (Morex_V2_scaf)’ datasets. Promoter sequences were screened using the ‘Binding Site Prediction’ tool from PlanRegMap (http://planttfdb.gao-lab.org/prediction.php) (Tian et al. 2019). Using the threshold *p*-value ≤ 1e^−4^, the lists of all TF binding sites in the promoter region were obtained (Supplementary Data 5).

To identify the TFs which possess significantly over-represented targets in DEG lists, previously obtained lists were analyzed with the ‘TF Enrichment’ tool from PlanRegMap (http://planttfdb.gao-lab.org/prediction.php) (Tian et al. 2019) using the following settings: species, *Hordeum vulgare*; method, motif; threshold *p*-value ≤ 0.05.

## Results

### Insensitivity to SLs affects shoot and root architecture in barley

**Fig. 1:**
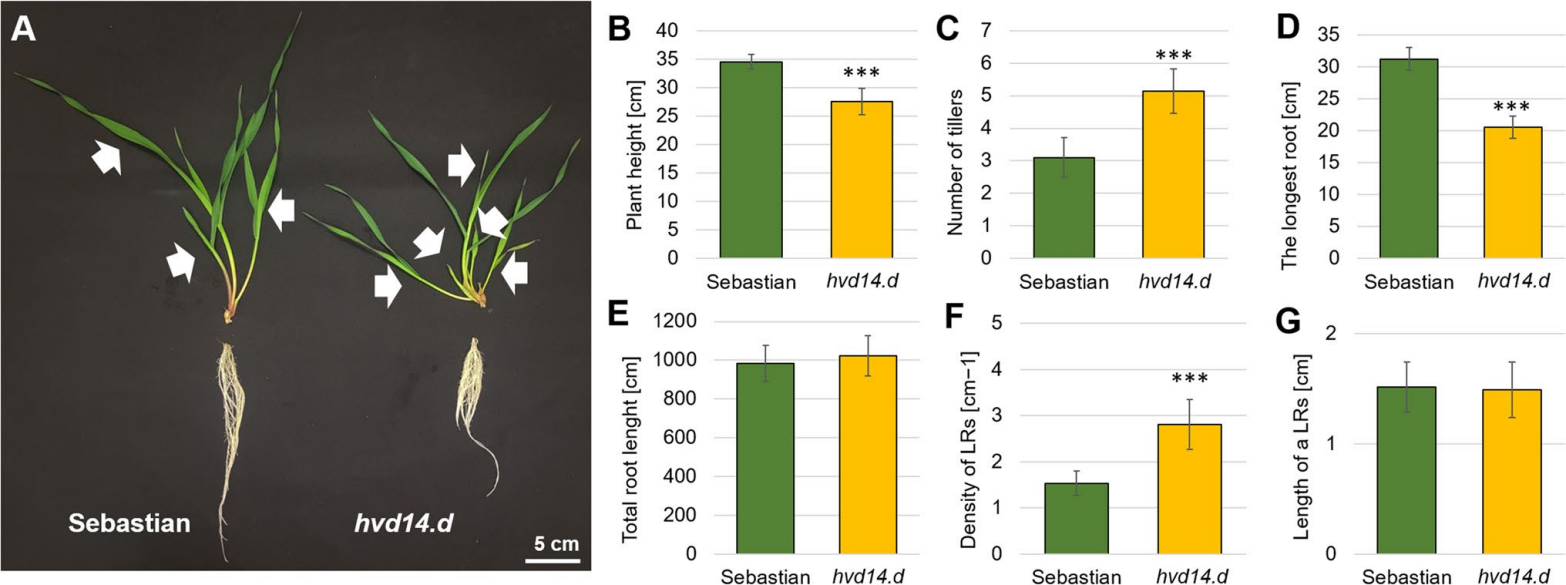
The phenotype of 3-week-old seedlings of Sebastian (wild-type) and *hvd14.d* (SL-insensitive mutant). **A** Differences in root and shoot between both genotypes. **B** Mutant *hvd14.d* exhibited a semi-dwarf phenotype and **C** produced significantly higher tillers than Sebastian. **D** Despite the shorter seminal roots of *hvd14.d*, the **E** total root length of both genotypes is similar. **F** Mutant *hvd14.d* developed more lateral roots than Sebastian, but **G** the length of lateral roots in both genotypes is similar. Asterisks indicate statistically significant differences between samples in a paired Student’s *t*-test (***correspond to *p*-values of *p* < 0.001; white arrows indicate tillers). LRs, lateral roots

Chemical mutagenesis and the TILLING strategy allowed the identification of a barley mutant with a mutation in the gene encoding the strigolactone receptor, *HvD14*. This mutation has been shown to change the conformation of the protein, narrowing the entrance to the active site, resulting in insensitivity to strigolactone. As reported previously, semi-dwarf barley mutant *hvd14.d* produces an almost two times higher number of tillers than the parent variety Sebastian (WT) when plants were grown in the soil (Marzec et al. 2016). Similar results were obtained for hydroponic conditions when comparing 3-week-old plants of both genotypes. The number of shoot branches in Sebastian (3.1 ± 0.61) was 40% lower than *hvd14.d* (5.1 ± 0.68). Additional assessment of phenotypic traits, the *hvd14.d* shoot height was 20% lower than in Sebastian (Fig. 1). The same number (seven) of seminal roots in both genotypes were observed, but the length of the longest seminal root was reduced in *hvd14.d* (65% of that noted for Sebastian). On the other hand, the total length of the root system in both genotypes was similar (984.9 ± 93.43 and 1023.3 ± 103.41 cm for Sebastian and *hvd14.d*, respectively) (Fig. 1). Those results can be explained by the more significant number of lateral roots in the mutant, which is in line with previous findings (Marzec et al. 2016). Indeed, under hydroponic conditions, 3-old-week *hvd14.d* plants exhibited a higher density of lateral roots than Sebastian. Still, the length of lateral roots was similar in both genotypes (1.5 ± 0.22 and 1.4 ± 0.25 cm for Sebastian and *hvd14.d*, respectively) (Fig. 1).

### Transcriptomic differences between Sebastian and hvd14.d

**Fig. 2:**
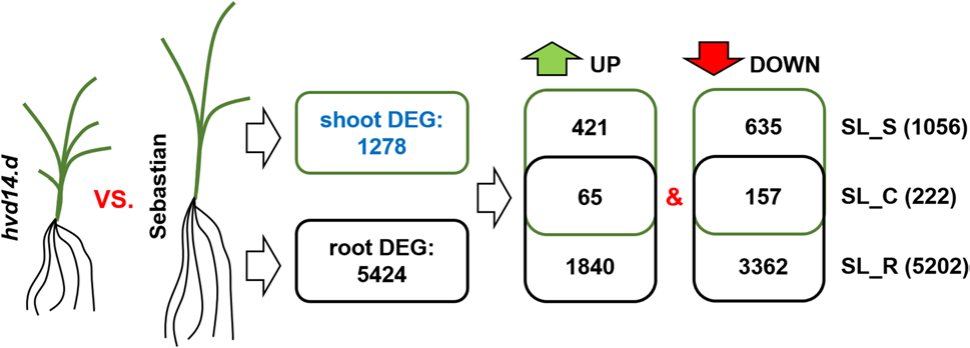
Overview of differentially expressed genes (DEGs) identified in shoot and root tissues when compared SL-insensitive barley mutant *hvd14.d* and its parent variety Sebastian (wild-type). SL_C—SL-related common genes; SL_S—SL-specific shoot DEGs; SL_R—SL-specific root DEGs

Gene expression was investigated separately for the shoot and root tissues of 3-week-old plants grown in hydroponics. A comparison of *hvd14.d* shoot transcriptome (d14_S) vs Sebastian shoot (Seb_S) revealed 1278 differentially expressed genes (DEG); 486 up, and 792 downregulated (adjusted *p*-value < 0.05 and |log_2_(FoldChange)| ≥ −1 or ≤ 1), while the comparison of root transcriptome (d14_R vs Seb_R) revealed an almost five times higher number of DEGs: 5424 (1905 up and 3519 downregulated) (Fig. 2, Supplementary Data 1). Analysis of these data revealed three sets of genes: (1) Genes differentially expressed in both shoot and root between genotypes were described as SL-related common genes (SL_C; 65 up, 157 downregulated), (2) SL-specific shoot DEGs (SL_S; 421 up, 635 downregulated) and (3) SL-specific root DEGs (SL_R; 1840 up, 3363 downregulated) when *hvd14.d* was compared to Sebastian (Fig. 2, Supplementary Data 1).

#### Non-organ-specific DEG analysis

Gene ontology (GO) enrichment analysis (FDR > 0.05) revealed that the SL_C upregulated set of genes is mainly related to RNA processing or metabolism (among biological process; BP) and RNA binding (among molecular function; MF), which is in agreement with the over-represented cellular component (CC) GO terms for those genes, which includes ribosome, nuclear or ribonucleoprotein complex localization. At the same time, downregulated SL_C genes were characterized as involved in photosynthesis, assimilation of photosynthetic products and response to light (BP). Consistent with these enriched BPs, enriched MF terms include chlorophyll-binding and the controlling activity of enzymes involved in sugar metabolism. In contrast, enriched CC terms include plastid, plastid membrane and photosystem localizations (Supplementary Data 2). The upper-hierarchy GO-terms revealed that DEGs common to shoot and root tissue may be involved in translation (upregulated) or photosynthesis, generation of precursor metabolites and energy (downregulated) (Supplementary Fig. 1; Supplementary Data 3).

#### Shoot-specific DEG analysis

Among the SL_S upregulated DEGs, enriched BP GO terms were related to RNA metabolism and processing, in addition to peptide biosynthesis and phosphorylation. Again, this is consistent with a ribosomal, mitochondrial and nuclear cellular localizations. Whereas downregulated SL_S DEGs were involved in cell wall organization and biosynthesis, enriched in an apoplast/cell wall and cytoskeletal cellular localization (Supplementary Data 2).

#### Root-specific DEG analysis

Lastly, we find that GO-enriched terms for roots were the most diverse, with upregulated SL_R DEGs enriched for peptide metabolism and response to various stimuli and stresses. Conversely, downregulated SL_R DEGs are enriched for genes involved in BP phosphorylation, cell communication, transport or response to the stimulus, while the CC-enriched terms included nuclear, plastidial or cell wall localizations (Supplementary Data 2). SL-dependent DEGs specific to only shoots or roots were more diverse regarding upper-hierarchy GO-terms. Moreover, those GO-terms do not coincide with those GO-terms described for the SL-dependent common DEG (Supplementary Fig. 1). This is another indication that the role of SLs in plant development depends on the type of tissue where they are active.

### Prediction of SL-related transcription factors (TF)

Given the substantial transcriptome changes we found in our study, we queried our dataset for potential transcription factors. We find that 6% (390) of the DEGs identified were TFs (Supplementary Data 4). The highest number of TFs was found among the SL_R (root-specific) DEGs, where we found 321 TFs. This result is related to the high number of Genes in this category because TF still account for 6% (321/5202), the same as in SL_C (common) (5.8%; 13/202) and SL_S (shoot-specific) (5.3%; 56/1056). By comparing Sebastian to *hvd14.d*, we can deduce which subset of these TFs may be related to SL signal transduction (TF_SL_DEG). For each 390 TF putatively involved in SL signalling, we identified the *A. thaliana* homologue (Supplementary Data 4), and within those homologues, six genes (AT1G09530, AT4G25560, AT2G02450, AT5G25190, AT3G16770, AT3G22830) were previously reported as SL-responsive (Wang et al. 2020).

Next, the promoter sequences (1500 bp) of each identified DEG were screened to find the TF binding sequences that regulate the transcription of those genes—TF_SL_PROM (Supplementary Data 5). This analysis showed that 65 identified above TF_SL_DEG recognize promoter binding sites in the DEG identified here (Supplementary Data 4). Finally, shoot and root DEG Lists were used to identify those TFs that are proposed to bind to the most represented promoter binding elements and therefore may regulate DEG expression. Those analyses allowed to type the 87 TF with significantly over-represented targets in DEG lists (30 – SL_C, 13 – SL_S, 44 – SL_R) (Supplementary Data 6).

Combining all previously described analyses, we were able to select 28 genes, which were (1) differentially expressed in *hvd14.d* vs Sebastian comparison (SL_DEG), (2) identified as TFs (TF_SL_DEG), (3) suggested to recognize TF motifs in the promoter region of other DEGs, and (4) motifs recognized by those TFs that are the most abundant among the DEG promoters (Table 1, Supplementary Data 7). No DEG from the SL_C category (differentially expressed in both shoot and root) was found among them. Whereas six and 22 TFs were found among shoot- and root-specific DEG populations, respectively. It has to be highlighted that all 28 TFs may recognize the targets that belong to each of the identified DEG categories: SL_C, SL_S and SL_R (Supplementary Data 7).

**Table 1:** Barley SL-dependent TFs which were identified in the presented study

	#	HORVU ID	MLOC ID	Best hit in A. thaliana	Description for the best hit	*TF_SL_DEG*	
						*hvd14.d* vs WT	
						log_2_FC	adj.pval
SL_S	**1**	**HORVU.MOREX.r2.1HG0041130.1**	MLOC_58950	AT4G17980.1	NAC domain-containing protein 71	2,17	1E−10
		TF involved in tissue reunion of wounded inflorescence stems; involved in the cellular response to auxin stimulus					
	**2**	**HORVU.MOREX.r2.7HG0557800.1**	MLOC_64612	AT1G51700.1	DOF zinc finger protein 1	−1,12	2E−03
		TF that binds specifically to a 5'-AA[AG] G-3’ consensus core sequence; involved in metal ion binding and response to chitin					
	**3**	**HORVU.MOREX.r2.6HG0479210.1**	MLOC_52112	AT5G11260.1	bZIP family protein	1,13	8E−13
		TF that promotes photomorphogenesis in light; involved in response to abscisic acid and response to karrikin					
	**4**	**HORVU.MOREX.r2.1HG0014470.1**	MLOC_59663	AT1G09540.1	myb domain protein 61	−3,33	4E−03
		Functions as a transcriptional regulator of stomatal closure; involved in vasculature development					
	**5**	**HORVU.MOREX.r2.1HG0046030.1**	MLOC_3095	AT2G44940.1	ERF family protein	−2,64	4E−07
		Involved in ethylene-activated signalling pathway					
	**6**	**HORVU.MOREX.r2.5HG0399940.1**	MLOC_70754	AT1G16060.1	ARIA-interacting double AP2 domain protein	−1,52	2E−03
		Involved in response to water deprivation and response to abscisic acid; positive regulation of the fatty acid biosynthetic process					
SL_R	**7**	**HORVU.MOREX.r2.2HG0148630.1**	MLOC_70754	AT5G17430.1	AP2 family protein	1,03	3E−06
		Regulation of transcription, DNA-templated					
	**8**	**HORVU.MOREX.r2.7HG0539480.1**	MLOC_70754	AT3G54320.1	AP2 family protein	−5,86	3E−04
		TF involved in response to sucrose; ethylene-activated signalling pathway; positive regulation of cutin biosynthetic process					
	**9**	**HORVU.MOREX.r2.6HG0499980.1**	MLOC_60958	AT2G02080.1	indeterminate(ID)-domain 4	−1,01	3E−13
		TF that may act as a transcriptional activator of nuclear-encoded photosynthetic gene expression					
	**10**	**HORVU.MOREX.r2.2HG0166600.1**	MLOC_66134	AT3G56400.1	WRKY DNA-binding protein 70	−1,32	2E−19
		TF involved in senescence, biotic and abiotic stress responses by modulating various phytohormones signalling pathways					
	**11**	**HORVU.MOREX.r2.3HG0230420.1**	MLOC_54606	AT1G29860.1	WRKY DNA-binding protein 71	−1,05	3E−10
		Regulation of transcription, DNA-templated					
	**12**	**HORVU.MOREX.r2.5HG0406860.1**	MLOC_10823	AT1G73730.1	ETHYLENE-INSENSITIVE3-like 3	−1,06	4E−10
		Encodes a putative TF involved in ethylene signalling					
	**13**	**HORVU.MOREX.r2.3HG0203860.1**	MLOC_68285	AT3G15030.2	TCP family protein	1,04	6E−03
		TF playing a pivotal role in the control of morphogenesis of shoot organs by negatively regulating the expression of boundary-specific genes					
	**14**	**HORVU.MOREX.r2.3HG0209060.1**	MLOC_68299	AT1G62300.1	WRKY family protein	−1,20	4E−23
		TF involved in response to low phosphate stress					
	**15**	**HORVU.MOREX.r2.4HG0342310.1**	MLOC_56769	AT2G18060.1	vascular-related NAC-domain protein 1	1,09	3E−03
		Expressed in root metaxylem pole and in shoot pre-procambium and procambium					
	**16**	**HORVU.MOREX.r2.3HG0236170.1**	MLOC_15725	AT3G26790.1	B3 family protein	2,78	1E−04
		Positive regulation of abscisic acid biosynthetic process					
	**17**	**HORVU.MOREX.r2.5HG0400770.1**	MLOC_13438	AT4G30980.1	LJRHL1-like 2	1,15	5E−15
		Involved in root hair elongation					
	**18**	**HORVU.MOREX.r2.6HG0471210.1**	MLOC_60890	AT1G80840.1	WRKY DNA-binding protein 40	−1,48	3E−27
		Involved in response to various phytohormones					
	**19**	**HORVU.MOREX.r2.5HG0371550.1**	MLOC_69575	AT1G13960.1	WRKY DNA-binding protein 4	−1,61	1E−26
		Involved in response to various phytohormones					
	**20**	**HORVU.MOREX.r2.4HG0315980.1**	MLOC_65745	AT3G03660.1	WUSCHEL-related homeobox 11	−3,49	3E−07
		TF involved adventitious root development					
	**21**	**HORVU.MOREX.r2.4HG0316110.1**	MLOC_14619	AT3G20770.1	EIL family protein	−1,34	8E−71
		TF involved in response to hypoxia					
	**22**	**HORVU.MOREX.r2.1HG0074290.1**	MLOC_6711	AT2G46270.1	G-box-binding factor 3	−1,16	3E−11
		Response to abscisic acid					
	**23**	**HORVU.MOREX.r2.3HG0207800.1**	MLOC_65876	AT2G20570.1	GBF’s pro-rich region-interacting factor 1	−1,96	3E−06
		Positive regulation of organelle organization					
	**24**	**HORVU.MOREX.r2.6HG0460660.1**	MLOC_63436	AT1G75390.1	basic leucine-zipper 44	1,06	3E−07
		Regulation of transcription, DNA-templated					
	**25**	**HORVU.MOREX.r2.1HG0023190.1**	MLOC_12079	AT5G26170.1	WRKY DNA-binding protein 50	−3,30	6E−21
		TF involved in jasmonic acid-mediated signalling pathway					
	**26**	**HORVU.MOREX.r2.5HG0366230.1**	MLOC_52439	AT5G59780.3	myb domain protein 59	1,29	4E−19
		Involved in response to various phytohormones					
	**27**	**HORVU.MOREX.r2.3HG0254080.1**	MLOC_67851	AT2G38470.1	WRKY DNA-binding protein 33	−2,22	1E−66
		TF involved in defense responses					
	**28**	**HORVU.MOREX.r2.2HG0079820.1**	MLOC_36657	AT5G13080.1	WRKY DNA-binding protein 75	−2,21	3E−18
		TF involved in lateral root development					

### Relational analysis of identified TFs using association networks

**Fig. 3:**
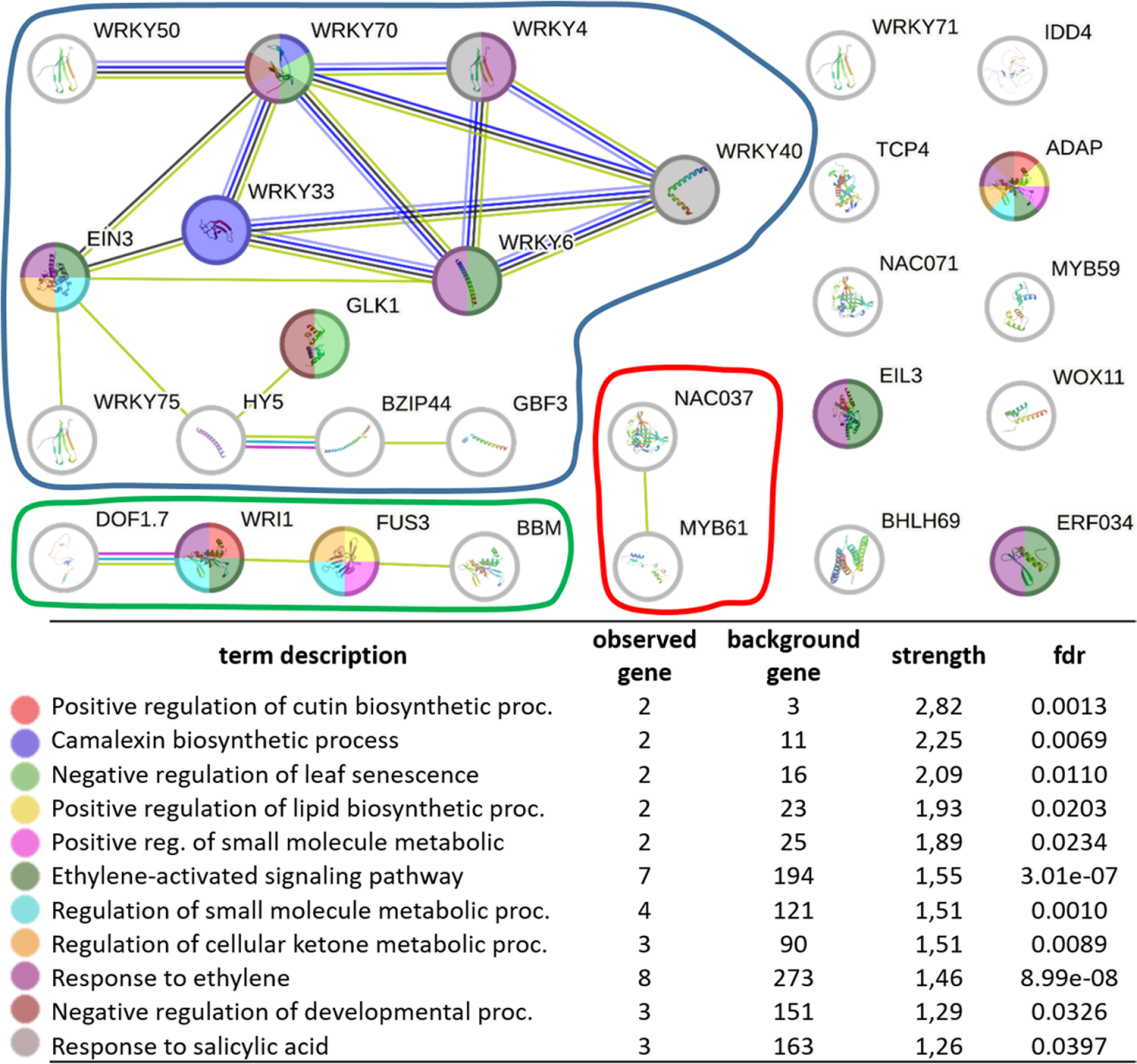
Protein-network analysis of SL-dependent TFs, performed using STRING Database. **A** Three networks of SL-dependent TFs of known or predicted interactions were identified. **B** Gene ontology enrichment analyses revealed the biological processes in which identified SL-dependent TFs might be involved; fdr, false discovery rate. Protein-protein interactions are presented as known interactions (experimentally determined: pink lines; from curated databases: light-blue line); predicted interactions (based on: gene co-occurrence: dark-blue; gene neighbourhood: dark-green), based on the co-expression (black) or text mining (light-green)

Next, to better contextualize our identified TFs, we used STRING-DB (Szklarczyk et al. 2023) to perform an association network analysis of the shoot and root TFs. We were able to link three groups of SL-dependent TFs, which interact with each other (Fig. 3, Supplementary Data 8). The largest network identified comprises 12 proteins (42% of all identified TFs), including seven TFs belonging to the WRKY family. GO analyses revealed that identified TFs are involved, i.e. in the regulation of cutin biosynthetic, camalexin biosynthesis, response to ethylene and salicylic acid, regulation of leaf senescence or lateral root development (Fig. 3, Supplementary Data 8).

### SLs induce expression of HORVU.MOREX.r2.1HG0041130.1 in HvD14-dependent manner

**Fig. 4:**
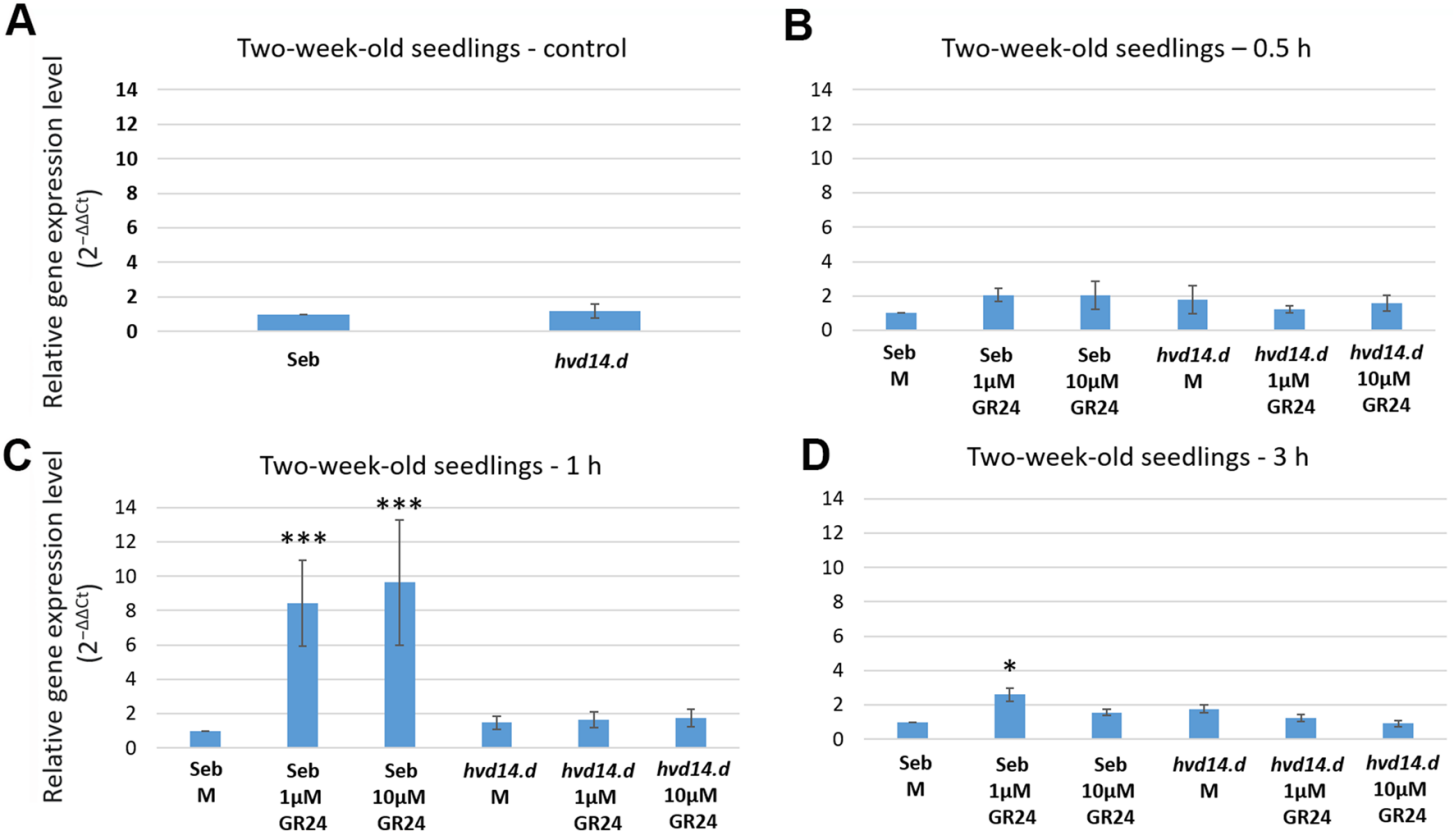
Analysis of HORVU.MOREX.r2.1HG0041130 Gene expression in tissues of 2-week-old seedlings of Sebastian and *hvd14.d* plants in response to GR24 treatment. **A** Relative level of expression of the HORVU.MOREX.r2.1HG0041130 gene in control (non-treated) plants **B** 30 min, **C** 1 h, and **D** 3 h after treatment with 1 plant treated with mock (0.01% acetone), 1 µM and 10 µM of GR24^5DS^. Statistical analyses were performed using the *t*-test (*p < .05; **p < .01; ***p < .001) comparing **A** *hvd14.d* vs Sebastian or SL-treated vs mock-treated plants. Mean value with standard deviation were presented

Among potential SL-dependent TFs which are active in barley shoots, the biggest difference in expression between *hvd14.d* and Sebastian (2.17 log_2_FC) was observed for HORVU.MOREX.r2.1HG0041130 (*A. thaliana* homologue: AT4G17980.1) (Table 1). In previous studies, the increased expression of this gene was observed in Sebastian (4.17 log_2_FC), but not in *d14*, during a response to drought (Daszkowska-Golec et al. 2023). To test the role of SLs in the control of HORVU.MOREX.r2.1HG0041130 expression, the SL spraying experiments on 2-week-old Sebastian and *hvd14.d* seedlings were performed. Two concentrations (1 and 10 µM) of synthetic SL analogue GR24 and a mock solution (0.01% acetone) were used. Before treatment, there were no differences in the expression of HORVU.MOREX.r2.1HG0041130 in the shoot of Sebastian and *hvd14.d* 2-week-old seedlings (Fig. 4). Thirty minutes after treatment, both SL concentrations do not alert the expression of HORVU.MOREX.r2.1HG0041130 in analyzed Genotypes compared to the control plants sprayed with the mock solution. However, 1 h after treatment, expression of the investigated Gene was induced by 1 and 10 µM of GR24 only in Sebastian. Finally, 3 h after treatment, induced expression was noted only in Sebastian seedlings sprayed with lower GR24 concentration (Fig. 4). The obtained data indicate that expression of HORVU.MOREX.r2.1HG0041130 is regulated in an HvD14-dependent manner because no effect of GR24 treatment was observed in *hvd14.d* plants. On the other hand, differences in HORVU.MOREX.r2.1HG0041130 expression observed for Sebastian seedlings at different times after treatment points out the temporal control of SLs on the expression of HORVU.MOREX.r2.1HG0041130.

## Discussion

### SL insensitivity impacts the development of barley shoot and root under hydroponic conditions

SLs are known mediators of shoot and root development, crucial in plant adaptation to environmental conditions. Photoperiod and temperature (Djennane et al. 2014), light intensity and length (Jia et al. 2014), and above all, nutrient availability (Yoneyama et al. 2013) affect SL biosynthesis/signalling, which influences plant development. Thus, the phenotype of SL mutants may vary depending on growing conditions. Here, we grew barley SL-insensitive mutant *hvd14.d* for the first time under hydroponic conditions. Previous analyses carried out on plants anchored in the soil or vermiculite revealed that *hvd14.d* produces twice as many tillers as WT. However, those differences were less pronounced in the first stages of plant development, i.e. 3-week-old *hvd14.d* plants grown in the soil produce 50% more tillers than Sebastian (3 ± 0.39 and 2 ± 0.21, respectively) (Marzec et al. 2016). Hydroponic culture in ½ Hoagland solution (Hothem et al. 2003) increased the number of tillers in both Genotypes at the same level. Still, the differences between Genotypes are similar, and a 60% higher number of tillers was observed in *hvd14.d* (Fig. 1). Plant height was the second feature differentiating both genotypes. The height of mutant *hvd14.d* grown in soil and hydroponics was reduced by about 20%. However, both genotypes were higher when grown in hydroponics (Sebastian: 34.5 ± 1.25 vs 29.1 ± 3.7 cm; *hvd14.d*: 27.5 ± 2.32 vs 22.4 ± 4.1 cm) (Marzec et al. 2016) (Fig. 1). It was previously reported that hydroponic conditions promote shoot development when compared to plants sown in soil (Dutta et al. 2023) that may be caused by easy access to water and nutrients in a hydroponic medium throughout development.

Under control conditions, SLs shape root architecture by promoting root elongation and inhibiting lateral root development (Kapulnik et al. 2011; Ruyter-Spira et al. 2011). Similar results were obtained when *hvd14.d* and Sebastian were grown in a solid medium (vermiculite) and watered with ½ MS medium. The mutant exhibited shorter seminal roots in a solid medium that produced more lateral branches than Sebastian (Marzec et al. 2016). Under hydroponic conditions, the same differences were observed (Fig. 1). In contrast, both genotypes in soil and hydroponic developed the same number of seminal roots, but their length was reduced, which is a well-known effect of hydroponics (Mian et al. 1993). Root elongation in the mutant was reduced by 35% under both conditions, hydroponic: 31.2 ± 1.79 vs 20.5 ± 1.74 cm and soil 47.1 ± 2.51 vs 34.8 ± 1.94 cm, respectively for Sebastian and *hvd14.d* (Marzec et al. 2016) (Fig. 1). Differences in lateral root density between genotypes were independent of growing conditions and *hvd14.d* developed a higher number of lateral roots per 1 cm of seminal root (Marzec et al. 2016) (Fig. 1). Obtained results indicate that growing conditions do not affect shoot and root differences between *hvd14.d* and its WT, as far as plants were supplemented with an optimal nutrient concentration.

### Tissue-specific transcriptome alterations caused by SL insensitivity

Because mutation in barley SL receptor HvD14 affects shoot and root development, those differences become statistically significant at the third week of plant development (Fig. 1). The transcriptome of shoot and root was compared between *hvd14.d* and Sebastian, revealing 6702 DEGs. Up to 80% of all identified DEGs were found in roots (5414 among 6702). In both organs, a more significant number of transcripts, around 60%, was downregulated (Fig. 2). Among all identified DEGs, only 3% (222) were found in both shoot and root comparisons. The largest category, up to 77% of all DEGs, was root-specific. These results indicate that SLs play a more pronounced role in root development relative to shoots in 3-week-old barley plants. It might also be related to the higher complexity of the root system at this stage of plant development, such as a large number of different cell types supporting vastly different transcriptional programs as is the case in *Arabidopsis* (Shahan et al. 2022). A 3-week-old barley seedling’s root system comprises seven seminal and hundreds of lateral roots at the different stages of development: initiation, elongation or branching. On the other hand, the shoot of a 3-week-old barley plant has from three to five tillers at the vegetative stage of development, which do not have developed internodes. Hence, more genes are involved in the development of the root, compared to the shoot, at this stage of plant growth. To date, there are no comparative studies about shoot and root transcriptomes for SL mutants, let alone a major crop species. Global analyses of transcriptome alterations caused by SL treatment or mutation in SL biosynthesis/signalling pathways were investigated for whole plants (Wang et al. 2020) or shoot/root separately (Zhan et al. 2018; Asghar et al. 2022; Haider et al. 2023; Li et al. 2023).

### SL-responsive genes found in barley shoot and root

In total, 222 DEGs were common between the shoot and root of *hvd14.d* compared to Sebastian. Among the 65 upregulated DEGs, 25% (16) were involved in translation along with others. Moreover, DEGs involved in ribosome biogenesis (4) or RNA processing (4) were found to be upregulated in *hvd14.d* (Supplementary Data 2). It was shown that SLs increase the cold tolerance of *Brassica rapa* L. seedlings because GR24 (a synthetic analogue of SL) treatment alleviates the damage of low-temperature stress (Zhang et al. 2020a, b). Within DEG between plants pretreated with spraying 0.1 μmol‧L^−1^ GR24 and non-pretreated, exposed to low temperature (4 °C), the genes encoding translation initiation factors were downregulated. On the other hand, in pea, removing apical meristem promotes the outgrowth of axillary buds, which was linked with increased expression (up to 35-fold) of gene encoding ribosomal protein (Stafstrom and Sussex 1992). The conclusion that SLs affect the translation processes via control of ribosome complex activity can be postulated. However, it cannot be excluded that stronger activity of the translational process observed in *hvd14.d* is related to the higher number of developing tillers and lateral roots, and those processes are associated with rapid protein synthesis. Hence, changes in expression of translation-related genes are not a direct result of SL activity, but the effect of SL insensitivity, resulting in the development of more meristems.

Surprisingly, both *hvd14.d* organs showed reduced gene expression related to photosynthesis and plastids (Supplementary Data 2). Changes in the expression of photosynthetic genes in non-green tissue, including roots have been widely reported for various species under different stresses, such as drought (Molina et al. 2008; Cohen et al. 2010; Ranjan and Sawant 2015; Janiak et al. 2019) or phosphate starvation (Wu et al. 2003; Li et al. 2010). It was shown that the suppression of photosynthetic genes is required for sustained root growth of *Arabidopsis* exposed to phosphorus deficit (Kang et al. 2014). Reduction in photosynthetic genes in roots during stress responses is also linked with decreased production of reactive oxidant species (ROS) (Kang et al. 2014; Janiak et al. 2019). Our previous analyses indicated that *hvd14.d* exhibits reduced ROS scavenging under drought (Daszkowska-Golec et al. 2023). Because SL treatment seems to decrease ROS content in various species (Trasoletti et al. 2022), including barley exposed to cadmium (Qiu et al. 2021), we may speculate that SL-insensitivity of *hvd14.d* results in less efficient ROS scavenging. Thus, to reduce ROS production, the mechanisms related to photosynthesis are repressed in SL-insensitive barley mutant under control conditions. In fact, study investigating the effect of SL on photosynthesis confirm these predictions. Treatment with 1 and 5 µM of GR24 increased the net photosynthesis rate (µmol CO_2_·m^−2^·S^−1^) of salt stressed rice seedlings to values observed in control plants (Ling et al. 2020). Further, in cucumber (*Cucumis sativus* L.), greater photosynthetic efficiency was observed in GR24-pretreated plants than in non-GR24-pretreated plants under salt stress (Zhang et al. 2022). Under control conditions, the foliar application of GR24 on *Artemisia annua* increased various attributes related to photosynthesis (chlorophyll fluorescence, internal CO_2_, and net photosynthetic rate) as well as activity of photosynthetic enzymes (carbonic anhydrase, nitrate reductase, RuBisCO) (Wani et al. 2023). The general positive role of SL on photosynthesis was well documented, so the decreased expression of photosynthesis-related genes in the shoot of SL-insensitive *hvd14.d* confirms these results. Conversely, repression of those genes in roots may be linked with reduction of processes linked to ROS production.

### Shoot- and root-specific SL-responsive genes

Within upregulated DEGs described as specific for shoot tissue, the largest group among the enriched GO terms was protein phosphorylation (Supplementary Data 2). Phosphorylation is one of the main post-translational modifications that affect protein interactions and stability, hence has a significant impact on gene expression, signalling pathways and enzyme activity (Khalili et al. 2022). Chen and co-workers indicated that GR24 treatment of rice SL-biosynthesis mutant (*d10*) changed the phosphorylation status of 8 proteins at a conserved phosphorylation site (Chen et al. 2014). Upregulated DEGs in *hvd14.d* involved in phosphorylation suggest that SLs may repress phosphorylation in barley shoots. On the other hand, among downregulated shoot DEGs, the large group was annotated as related to the cell wall organization and biogenesis, cell wall polysaccharide metabolic processes or polysaccharide biosynthetic and metabolic processes (Supplementary Data 2). There is a known role for SLs in promoting secondary cell wall formation in cotton (*Gossypium hirsutum*) where exogenous GR24 increased, and the application of SL biosynthetic inhibitor (Tis108) reduced the thickness of the secondary cell wall (Wen et al. 2023). Moreover, SL biosynthesis genes (*MAX3* and *MAX4*) have been linked with xylan and cellulose deposition in *Arabidopsis* (Ramírez and Pauly 2019). Further, we previously reported the alteration of cell wall formation in *hvd14.d* in response to drought (Marzec et al. 2020). Interestingly, this is a conditional phenomenon as under control conditions; there are no differences in the cell wall thickness in the leaves of 3-week-old seedlings of *hvd14.d* and Sebastian (Marzec et al. 2020); however, there have been no investigations into the chemical composition of the cell wall to date. Thus, the differences in the polymer content between *hvd14.d* and Sebastian cannot be excluded. Secondary cell walls contain mainly cellulose, xylans and lignin, but their proportions and modifications depend on the functional needs of cell/tissue and, thus may vary between leaves and roots (Kumar et al. 2016). The data obtained, where decreased expression of genes related to cell wall biosynthesis was found specifically in barley shoot (Supplementary Data 2), narrowed down the possible role of SLs in the biosynthesis of cell wall components to that characteristic for shoot.

Within SL-related upregulated DEG found in roots, a significant number was annotated as cell cycle or cell cycle processes. It could be explained by the larger number of developing roots, thus the higher number of fast-dividing meristems in *hvd14.d* compared to the Sebastian (Fig. 1). On the other hand, both up and downregulated DEGs were annotated as involved in responses to abiotic stresses, stimuli and chemical or oxygen-containing compounds. Because SLs play an important role in plant adaptation to stresses, the insensitivity to SLs may disturb the multiple pathways related to the plant’s stress responses.

### SL-dependent TFs

General knowledge of the SL signalling pathway and the individual proteins involved in signal transduction is well established in model species such as *Arabidopsis* or rice, from the SL signal perception to the degradation of the SL repressor (Marzec and Brewer 2019). However, we still have rudimentary information about the transcriptional responses in crops and non-model plants. Particularly the TFs that regulate the plant’s response to SLs. Here, by simultaneously comparing changes in the shoot and root transcriptome of *hvd14.d* and Sebastian, we proposed a set of TFs that may play a role in SL signal transduction in barley and which are involved in phenotypic changes observed in the shoot and root architecture of 3-week-old plants described above. In total, 28 TFs were identified as putative SL-related TFs as they (1) exhibit changed expression in *hvd14.d* versus Sebastian, (2) they are proposed to recognize binding sites in promoters of a multitude of identified DEGs and (3) motifs recognized by those TFs are over-represented (*p*-value ≤ 0.05) in DEG promoters (Table 1, Supplementary Data 9).

Interestingly, no one TF was differentially expressed in shoot and root barley tissue (SL_C), indicating differences exist in SL signal transduction between these two organs. Four TFs were previously identified as putatively involved in mediated SL-dependent barley response to drought (Daszkowska, 2023) (Supplementary Fig. 2). Two of these HORVU.MOREX.r2.6HG0471210.1 (AT1G80840) and HORVU.MOREX.r2.1HG0074290.1 (AT2G46270) are involved in plant response to abscisic acid (ABA) and were found to be upregulated by drought only in the Sebastian shoot. At the same time, under control conditions, their expression was downregulated in *hvd14.d* root relative to Sebastian (Daszkowska-Golec et al. 2023). It was previously shown that ABA may regulate lateral root formation (De Smet et al. 2003; Orman-Ligeza et al. 2018). However, the interactions between SLs and ABA have been described in various aspects of plant development under both control and stress conditions (Korek and Marzec 2023). Thus, we may conclude that the higher number of lateral roots observed in *hvd14.d* is related to the disorder in ABA signalling caused by the SL-insensitivity, similar to a weaker response of *hvd14.d* to drought stress (Daszkowska-Golec et al. 2023).

Another TF, HORVU.MOREX.r2.1HG0041130.1 (AT4G17980) mediates the auxin response and was upregulated in *hvd14.d* shoots. Auxin export, which is necessary for the outgrowth of axillary buds, is blocked by SLs to suppress shoot branch development (Shinohara et al. 2013). In *hvd14.d*, which develops a higher number of tillers, the increased auxin export induces auxin signalling, i.e. via expression of HORVU.MOREX.r2.1HG0041130.1. The last TF identified as SL-dependent under control and drought conditions was HORVU.MOREX.r2.3HG0209060.1 is an ortholog of WRKY6 in *Arabidopsis* (AT1G62300) and is described as being involved in response to low phosphate (Chen et al. 2009). Under phosphorus deficiency, WRKY6 binds the promoter of PHOSPHATE1 (PHO1) (Chen et al. 2009), increasing the production of lateral roots (Williamson et al. 2001). Given the observed root phenotype observed here, HORVU.MOREX.r2.3HG0209060.1 could play a broader role in SL-dependent repression of lateral root development in barley. Because SL treatment induced HORVU.MOREX.r2.1HG0041130 expression in WT plant, but not in the SL-insensitive mutant *d14* (Fig. 4), we may assume that SLs control HORVU.MOREX.r2.1HG0041130 expression in a D14-dependent manner. Moreover, it was also previously shown that in response to drought, the expression of HORVU.MOREX.r2.1HG0041130 increases in Sebastian plants, but not in the *d14* mutant (Daszkowska-Golec et al. 2023)*.* On the other hand, exogenous GR24 induced the HORVU.MOREX.r2.1HG0041130 expression 1 (1 and 10 µM) or 3 h (1 µM) after treatment, but not after 30 min (Fig. 4). Thus, the temporal control of SLs on HORVU.MOREX.r2.1HG004113 can be postulated, which also depends on the SL concentration. Because, in older plants (3-week-old plants) grown in hydroponics, the increased expression of HORVU.MOREX.r2.1HG0041130 was observed in *d14*; the open question remains how plant developmental stage and growing conditions affect the expression of HORVU.MOREX.r2.1HG0041130

Assessment of the association between identified SL-dependent TFs revealed significantly more interactions than expected (PPI enrichment *p*-value:< 1.0e−16), indicating that the proteins are at least partially biologically connected. Moreover, 42% of all identified SL-dependent TFs were grouped in the single network of known and predicted interactions (Fig. 3). As expected, among all TFs, the proteins annotated as hormonal responsive were overrepresented. However, proteins involved in response to ethylene and salicylic acid were also identified, pointing out the interactions between SLs and those two phytohormones in shaping shoot and root architecture in barley. Finally, two out of three genes related to positive regulation of cutin biosynthesis were identified as SL-dependent TFs (Fig. 3). Cutin is a main component of the cuticle (Fich et al. 2016), with the biosynthesis pathway similar to other plant hydrophobic polymer suberin (Pollard et al. 2008) that accumulates in the apoplastic regions of non-cutinized boundary cell layers, such as root exodermis (Vishwanath et al. 2015). Previously, it was postulated that SLs modulate wax biosynthesis and deposition in plants (Li et al. 2020b; Marzec et al. 2020; Li et al. 2019).

Interestingly, genes controlling camalexin biosynthesis were found among SL-dependent TFs. Camalexin is one of the phytoalexins, which are the antimicrobial compounds produced by plants (Hammerschmidt 1999). SLs may play a dual role in interactions with bacteria and fungi to (1) promote the symbiosis with arbuscular mycorrhizal (AM) fungi (Kodama et al. 2022) or (2) increase the resistance against pathogen bacteria and fungi (Marzec 2016). Thus, it may be postulated that SLs control microbial interactions via camalexin synthesis. However, a new role of camalexin in controlling lateral root formation in *Arabidopsis* was recently described (Serrano-Ron et al. 2021). Up to now, a similar function of camalexin in monocots has not been reported. Still, it cannot be excluded that SL-insensitivity in barley disturbs camalexin biosynthesis, which affects lateral root development.

## Supplementary Information

Below is the link to the electronic supplementary material.

Supplementary file1 (XLSX 789 KB)

Supplementary file2 (XLSX 28.0 KB)

Supplementary file3 (XLSX 19.0 KB)

Supplementary file4 (XLSX 44.6 KB)

Supplementary file5 (XLSX 55.2 MB)

Supplementary file6 (XLSX 15.3 KB)

Supplementary file7 (XLSX 15.9 KB)

Supplementary file8 (XLSX 14.3 KB)

## Data Availability

The data underlying this article are available in the article and its online Supplementary material. Transcriptomic data are available in the ArrayExpress repository: E-MTAB-13641.
